# Mitigation of a nitrate reducing *Pseudomonas aeruginosa* biofilm and anaerobic biocorrosion using ciprofloxacin enhanced by D-tyrosine

**DOI:** 10.1038/s41598-017-07312-7

**Published:** 2017-07-31

**Authors:** Ru Jia, Dongqing Yang, Dake Xu, Tingyue Gu

**Affiliations:** 1 Department of Chemical and Biomolecular Engineering, Institute for Corrosion and Multiphase Technology, Ohio University, Athens, OH 45701 USA; 2 School of Materials Science and Engineering, Northeastern University, Shenyang, 110819 China

## Abstract

*Pseudomonas aeruginosa* (PA) is a ubiquitous microbe. It can form recalcitrant biofilms in clinical and industrial settings. PA biofilms cause infections in patients. They also cause biocorrosion of medical implants. In this work, D-tyrosine (D-tyr) was investigated as an antimicrobial enhancer for ciprofloxacin (CIP) against a wild-type PA biofilm (strain PAO1) on C1018 carbon steel in a strictly anaerobic condition. Seven-day biofilm prevention test results demonstrated that 2 ppm (w/w) D-tyr enhanced 30 ppm CIP by achieving extra 2-log sessile cell reduction compared with the 30 ppm CIP alone treatment. The cocktail of 30 ppm CIP + 2 ppm D-tyr achieved similar efficacy as the 80 ppm CIP alone treatment in the biofilm prevention test. Results also indicated that the enhanced antimicrobial treatment reduced weight loss and pitting corrosion. In the 3-hour biofilm removal test, the cocktail of 80 ppm CIP + 5 ppm D-tyr achieved extra 1.5-log reduction in sessile cell count compared with the 80 ppm CIP alone treatment. The cocktail of 80 ppm CIP + 5 ppm D-tyr achieved better efficacy than the 150 ppm CIP alone treatment in the biofilm removal test.

## Introduction

The colonization and infection by microbes lead to chronic wounds which are common in individuals^1^. It was reported that *Pseudomonas* species are commonly isolated bacteria in chronic wounds^2^. Within wounds, bacteria predominantly exist as biofilms^3^. Biofilms are communities of microorganisms surrounded by extracellular polymeric substances on a surface^4, 5^. They cause infections^6^, biofouling^7^, and biocorrosion^8, 9^. Biofilms protect sessile cells from antimicrobial agents using several mechanisms such as diffusional limitation, lowered metabolic rates, formation of persister cells, upregulation of resistance genes, and efflux pumps^10^.

*Pseudomonas* species are ubiquitous in natural environments and clinical settings^11, 12^. *Pseudomonas aeruginosa* (PA) is a Gram-negative bacterium that can form persistent biofilms on catheters, contact lenses, and cystic fibrosis (CF) infected lungs^13^. This opportunistic pathogen can be life-threatening to CF patients^14^. PA biofilms in CF lungs grow anaerobically^15^. The biofilms infecting medical implants are usually anaerobic^16^. In anaerobic respiration, PA can use nitrate and nitrite as terminal electron acceptors^15^. PA biofilms also cause corrosion of carbon steel and stainless steel in various environments^17, 18^. This kind of corrosion caused by biofilms on metal surfaces, are known as biocorrosion^19^. Stainless steel is a biocompatible metal which is often used in biomedical implants such as orthopedic and dental implants^20^. Although stainless steel is corrosion-resistant, it is subject to varying degrees of biocorrosion weight loss and pitting damages^20, 21^.

Usually 10 times or higher antimicrobial concentrations are required to treat sessile cells in biofilms than that needed to treat planktonic cells^22^. In clinical settings, drug-resistance is encountered because of the excessive use of antibiotics^23^. The high concentration dosing over time may lead to resistance that causes dosage escalation. Therefore, a more effective antimicrobial treatment is desirable.

Antimicrobial enhancers can work synergistically with existing antimicrobials^24^. D-amino acids are natural chemicals that have been found to disperse *Bacillus subtilis* and *Staphylococcus aureus* biofilms^25^. D-amino acids are found in some protein-rich food products because of the conversion of some L-amino acids to D-amino acids under heat and alkaline pH during food processing^26^. It was also found that D-amino acids alone did not inhibit the PA biofilm formation^27^. Therefore, an antibiotic stress is needed together with D-amino acids. Several antibiotics (amikacin, colistin, ciprofloxacin, imipenem, and ceftazidime) were found to be enhanced by D-methionine, D-phenylalanine, D-tryptophan, and their equimolar mixture against the aerobic PA biofilms in polystyrene 96-well plates^11^. The dosages of D-amino acids used were more than 500 ppm^11^. In our previous study, 1 ppm D-tyrosine (D-tyr) enhanced biocides (tetrakis (hydroxymethyl) phosphonium sulfate (THPS) and alkyldimethylbenzylammonium chloride (ADBAC)) against a *Desulfovibrio vulgaris* biofilm (a corrosive sulfate reducing bacterium biofilm) on carbon steel coupons under an anaerobic condition^28, 29^.

In this work, D-tyr at a low concentration (2–5 ppm) was evaluated as an antimicrobial enhancer for ciprofloxacin (CIP) against the wild-type *P. aeruginosa* (PAO1) biofilm on C1018 carbon steel in an anaerobic environment. CIP is a broad-spectrum antibiotic^11^. The combination of CIP and D-tyr against the anaerobic PA biofilm has never been reported before. There are quite a few reports in the literature on biocorrosion by aerobic PA biofilms^9, 30, 31^, but very few on biocorrosion by anaerobic PA biofilms grown as nitrate reducers^32^. The objective of this research was to show that D-tyr at a low concentration reduced CIP dosage while achieving better efficacies in the prevention of the anaerobic PA biofilm and the removal of established anaerobic PA biofilm. Instead of stainless steel, carbon steel was used in this work to grow a much more recalcitrant PA biofilm in a short time (7 days) in the laboratory.

## Results

### Sessile cell counts and weight losses in the PA biofilm prevention test

**Figure 1:**
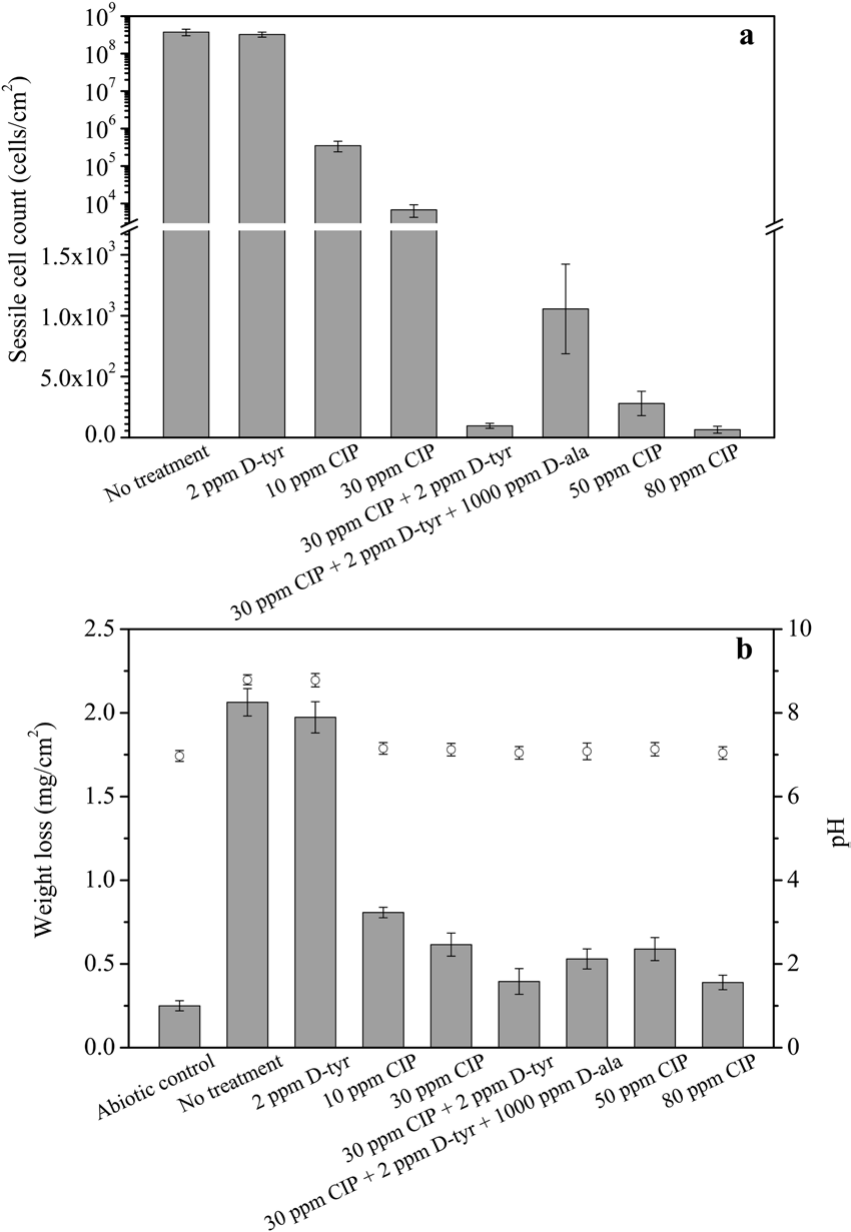
Sessile cell counts, specific weight losses and pH. (**a**) Sessile cell counts after the 7-day biofilm prevention test with different treatment chemicals in the culture medium. (Error bars represent standard deviations, statistical reference point n = 4). (**b**) Weight losses of coupons (bars) and pH values (circles) of the culture medium at the end of the 7-day biofilm prevention test. (Error bars represent standard deviations, statistical reference point n = 6).

Figure 1a shows the PA sessile cell counts on carbon steel coupons after the 7-day biofilm prevention test with different treatments. The sessile cell count on the no treatment control coupon was 3.8 × 10^8^ cells/cm^2^. Two ppm D-tyr alone treatment didn’t show any log reduction of the sessile cell count compared with the no treatment control. Ten ppm CIP alone treatment and 30 ppm CIP alone treatment reduced the sessile cell counts to 3.5 × 10^5^ cells/cm^2^ and 9.1 × 10^3^ cells/cm^2^, respectively. Fifty ppm CIP alone treatment and 80 ppm CIP alone treatment reduced the sessile cell counts to 3.1 × 10^2^ cells/cm^2^ and less than 1.0 × 10^2^ cells/cm^2^, respectively. The cocktail of 30 ppm CIP + 2 ppm D-tyr also reduced the sessile cell count to less than 1.0 × 10^2^ cells/cm^2^ by achieving extra 2-log reduction in sessile cell count compared with the 30 ppm CIP alone treatment. The sessile cell count with the treatment of 30 ppm CIP + 2 ppm D-tyr was lower than that in the 50 ppm CIP alone treatment. The combination of 30 ppm CIP + 2 ppm D-tyr led to a significant decrease in sessile cell amount compared with the 30 ppm CIP alone (p < 0.001) and the 50 ppm CIP alone (p = 0.002) treatments. Results here showed that D-tyr enhanced the low concentration of CIP by achieving a similar efficacy as the high concentration of CIP because statistically, the sessile cell count for the combination of 30 ppm CIP + 2 ppm D-tyr was not significantly different from the 80 ppm CIP alone treatment (p = 0.11). Although 1 ppm D-tyr could enhance THPS or ADBAC against the *D. vulgaris* biofilm by achieving at least an extra 2-log sessile cell count reduction in the 7-day *D. vulgaris* biofilm prevention test^28, 29^. Here 1 ppm D-tyr was found not sufficient to enhance CIP (data not shown) in the PA biofilm prevention test. This suggested that the PA biofilm was tougher. Thus, 2 ppm D-tyr was used here. The combination of 30 ppm CIP + 2 ppm D-tyr + 1000 ppm D-ala led to an increase in the sessile cell amount compared with the combination of 30 ppm CIP + 2 ppm D-tyr.

The abiotic test was carried out to verify the effect of treatment chemicals in the absence of microbes on the coupon weight loss in an anaerobic condition. The 125 ml abiotic vial contained 100 ml culture medium with 100 ppm L-cysteine, 80 ppm CIP and 2 ppm D-tyr. The weight loss of the abiotic control in Fig. 1b shows that the chemicals in the culture medium had negligible effect on the coupon weight loss. The specific weight losses for the no treatment control and 2 ppm D-tyr alone treatment were 2.06 ± 0.09 mg/cm^2^ and 1.97 ± 0.08 mg/cm^2^, respectively. The D-tyr alone treatment showed no significant weight loss reduction. The weight loss decreased when the concentration of CIP was increased from 30 ppm to 80 ppm. The cocktail of 30 ppm CIP + 2 ppm D-tyr and the 80 ppm CIP alone treatments showed similar weight losses indicating that D-tyr reduced the CIP dosage considerably. The combination of 30 ppm CIP + 2 ppm D-tyr + 1000 ppm D-ala led to a higher weight loss compared with the combination of 30 ppm CIP + 2 ppm D-tyr.

Statistically, the combination of 30 ppm CIP + 2 ppm D-tyr yielded a significant decrease in weight loss compared with the 30 ppm CIP alone (p = 0.001) and the 50 ppm CIP alone (p = 0.002) treatments. The weight loss for the combination of 30 ppm CIP + 2 ppm D-tyr treatment was not significantly different from the 80 ppm CIP alone treatment (p = 0.44). The weight loss data here are consistent with the sessile cell counts in Fig. 1a. The pH values for all treatments in the media after the 7-day incubation were all above 7. The PA biofilm was a nitrate reducing bacterium (NRB). The typical NRB biofilm corrosion is caused by the utilization of the electrons from elemental iron oxidation for nitrate reduction^33^. The pH data eliminated the possibility of organic acid corrosion that is significant only at a much lower pH.

### Biofilm observations in the PA biofilm prevention test

**Figure 2:**
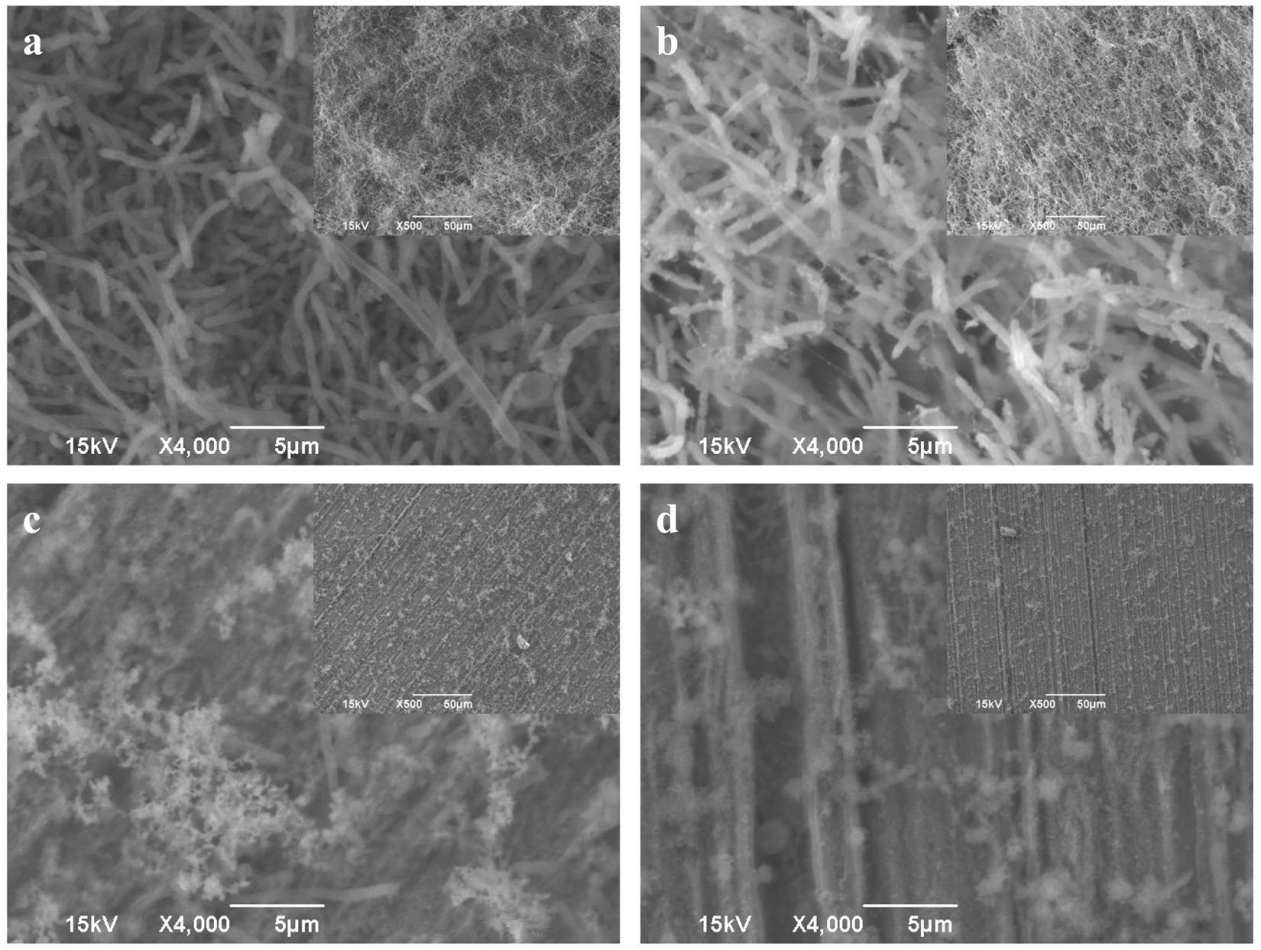
SEM images of PA biofilms on C1018 after the 7-day biofilm prevention test with: (**a**) no treatment (control), (**b**) 2 ppm D-tyr, (**c**) 30 ppm CIP, and (**d**) 30 ppm CIP + 2 ppm D-tyr. (Scale bars in the small inserted images are 50 μm).

Figure 2 shows the scanning electron microscope (SEM) images of the PA biofilms on the surfaces of coupons after different treatments in the 7-day biofilm prevention test. Abundant sessile cells were found on the no treatment control (Fig. 2a). When 2 ppm D-tyr alone was used (Fig. 2b), the sessile cells were still abundant. This indicates that the D-tyr alone was insufficient to prevent the PA biofilm formation. Sessile cells were still easily found on the coupon surface when 30 ppm CIP alone was used (Fig. 2c). The cocktail of 30 ppm CIP + 2 ppm D-tyr led to much fewer sessile cells in Fig. 2d. The SEM biofilm observations were consistent with the sessile cell counts in Fig. 1a and the weight loss data in Fig. 1b. Although SEM images show cell morphology and exopolymeric substance (EPS) clearly, they cannot tell the difference from dead cells and live cells.

**Figure 3:**
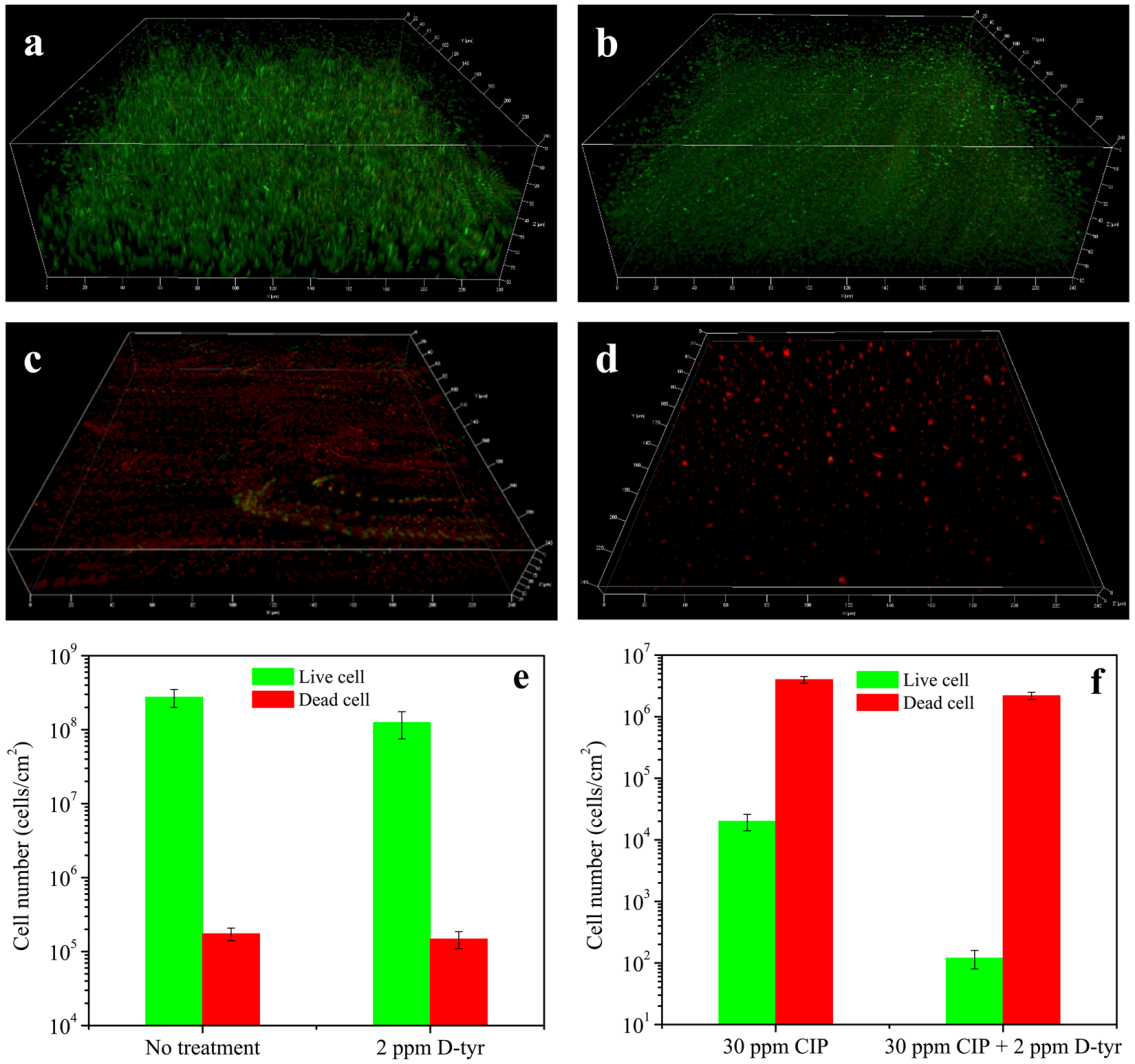
CLSM images of PA biofilms (Dimensions: X = 224 µm, Y = 224 µm) on C1018 after the 7-day biofilm prevention test with: (**a**) no treatment (control) (biofilm thickness = 76 µm), (**b**) 2 ppm D-tyr (biofilm thickness = 74 µm), (**c**) 30 ppm CIP (biofilm thickness = 34 µm), and (**d**) 30 ppm CIP + 2 ppm D-tyr (biofilm thickness = 10 µm). The calculated numbers of live/dead cells are shown in (**e**) and (**f**). (Error bars represent standard deviations, statistical reference point n = 6).

Figure 3 shows the confocal laser scanning microscopy (CLSM) images of the PA biofilms on the coupon surfaces with different treatments after the 7-day biofilm prevention test. Fig. 3(a,b) indicates that live cells (green dots) were abundant on the no treatment control and the 2 ppm D-tyr alone treatment coupon surfaces. A lot of sessile cells were killed (red dots) when the 30 ppm CIP alone treatment was used, but there were still some live cells (Fig. 3c). With the cocktail of 30 ppm CIP + 2 ppm D-tyr (Fig. 3d), dead cells were abundant while live cells were hardly found. Figure 3e and f show the numbers of live and dead sessile cells for the no treatment control, 2 ppm D-tyr, 30 ppm CIP, and 30 ppm CIP + 2 ppm D-tyr treatments after the 7-day biofilm prevention test. They indicate that D-tyr alone had a limited effect on biofilm formation. The combination of 30 ppm CIP + 2 ppm D-tyr showed better efficacy than the 30 ppm CIP alone treatment. Statistically, the combination produced a significant decrease in sessile cell amount compared with the 30 ppm CIP alone treatment (p = 0.001). CLSM images in Fig. 3 corroborated the sessile cell counts in Fig. 1a, the weight loss data in Fig. 1b, and the SEM images in Fig. 2. The biofilm observations using SEM and CLSM clearly indicate that D-amino acid worked synergistically with CIP. It also confirmed that an antimicrobial stress is necessary for D-amino acids to disperse recalcitrant biofilms^24^.

### Pit morphology and profile in the PA biofilm prevention test

**Figure 4:**
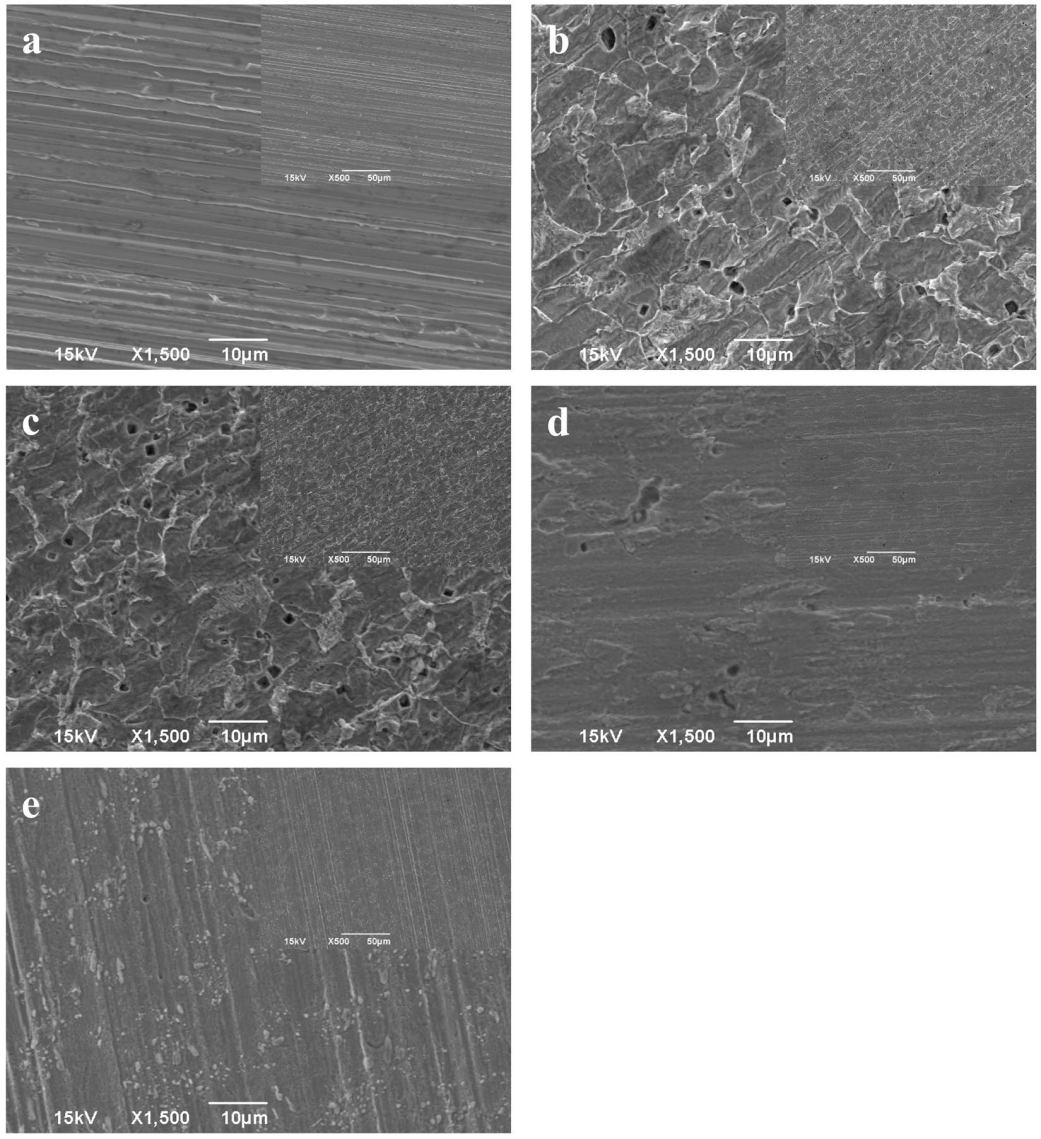
SEM pit images on coupon surfaces after the 7-day biofilm prevention test with: (**a**) abiotic control, (**b**) no treatment, (**c**) 2 ppm D-tyr, (**d**) 30 ppm CIP, and (**e**) 30 ppm CIP + 2 ppm D-tyr. (Scale bars in the small inserted images are 50 μm).

Figure 4 shows the pit images on the coupon surfaces for the abiotic control, the no treatment control, the 2 ppm D-tyr alone treatment, the 30 ppm CIP alone treatment, and the 30 ppm CIP + 2 ppm D-tyr cocktail treatment after the 7-day biofilm prevention test. Figure 4a confirms that the treatment chemicals were not corrosive. Obvious pits were found on the no treatment control (Fig. 4b) and the 2 ppm D-tyr alone (Fig. 4c) coupon surfaces. The cocktail of 30 ppm CIP + 2 ppm D-tyr (Fig. 4e) led to much fewer pits compared with the 30 ppm CIP alone treatment (Fig. 4d). The pit images are consistent with the weight loss data in Fig. 1b.

**Figure 5:**
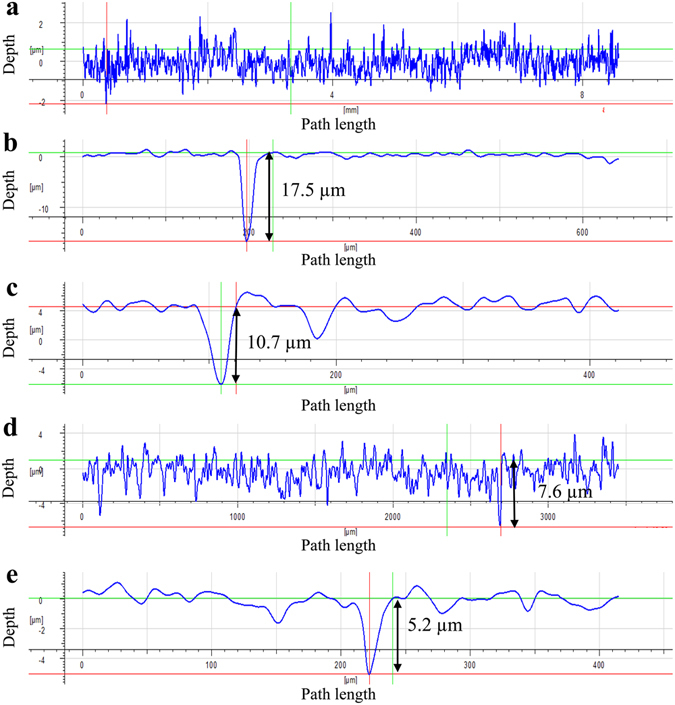
IFM pit depth profile of coupons after the 7-day biofilm prevention test with: (**a**) abiotic control, (**b**) no treatment, (**c**) 2 ppm D-tyr, (**d**) 30 ppm CIP, and (**e**) 30 ppm CIP + 2 ppm D-tyr. (Scale bars in the small inserted images are 50 μm).

The maximum pit depth data of coupons with different treatments after the 7-day biofilm prevention test are shown in Fig. 5. Figure 5a confirms that the abiotic control coupon did not show any pitting. The maximum pit depths for the no treatment control, the 2 ppm D-tyr alone treatment, the 30 ppm CIP alone treatment, and the 30 ppm CIP + 2 ppm D-tyr cocktail treatment were 17.5 µm, 10.7 µm, 7.6 µm, and 5.2 µm, respectively. Results showed that the cocktail of 30 ppm CIP + 2 ppm D-tyr reduced the maximum pit depth more than the 30 ppm CIP alone treatment. The maximum pit depth data of different treatments in Fig. 5 are consistent with the pit images in Fig. 4.

### Sessile cell counts in the PA biofilm removal test

**Figure 6:**
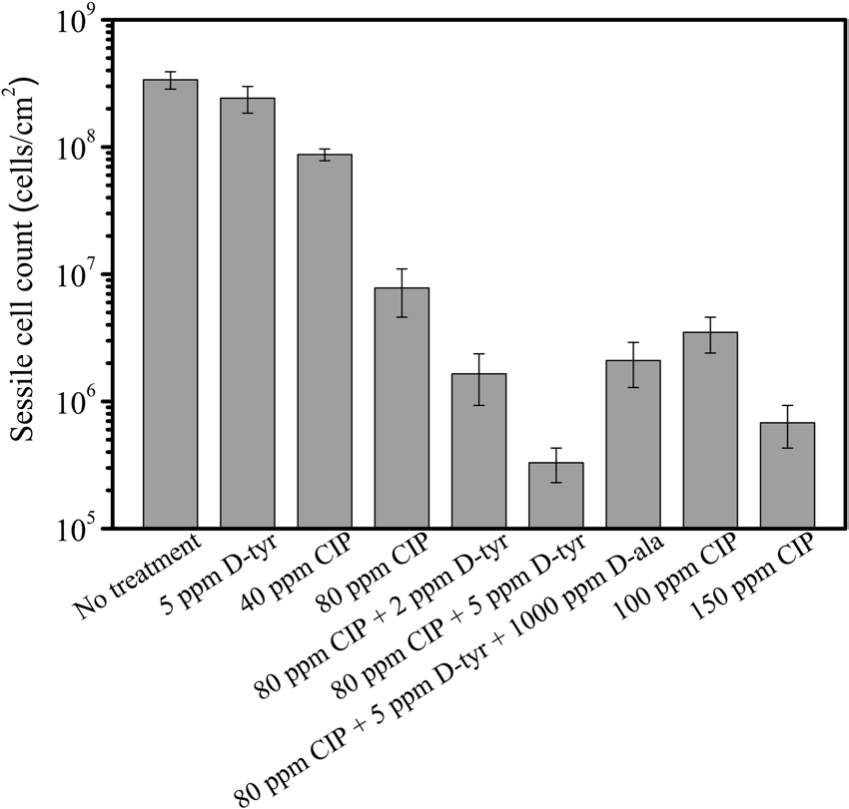
Sessile cell counts after the 3-hour biofilm removal test. (Error bars represent standard deviations, statistical reference point n = 4).

In this test, biofilms were pre-grown for 2 days to achieve maturity on the coupon surfaces. After that, different treatments were applied to the biofilms in the anaerobic chamber for 3 hours. Figure 6 shows the sessile cell counts with different treatments. After the 3-hour treatment, 5 ppm D-tyr alone did not achieve any log reduction of the sessile cell count compared with the no treatment control. The same was true for the 40 ppm CIP alone treatment. When the CIP concentration increased to 80 ppm, a 1.5-log reduction of sessile cell count was achieved compared with the no treatment control. A 2-log reduction of the sessile cell count was achieved when CIP concentration increased further to 150 ppm. The cocktail of 80 ppm CIP + 2 ppm D-tyr did not show any extra log reduction of sessile cell count compared with the 80 ppm CIP alone treatment. However, when the concentration of D-tyr increased to 5 ppm, the cocktail of 80 ppm CIP + 5 ppm D-tyr achieved extra 1.5-log reduction in sessile cell count compared with the 80 ppm CIP alone treatment. The efficacy of the cocktail of 80 ppm CIP + 5 ppm D-tyr was better than the 150 ppm CIP alone treatment. Statistically, the cocktail of 80 ppm CIP + 5 ppm D-tyr yielded a significant decrease in sessile cell amount compared with the 80 ppm CIP alone (p = 0.003), the 100 ppm CIP alone (p = 0.002), and the 150 ppm CIP alone (p = 0.01) treatments. The data here show that 5 ppm D-tyr was an effective antimicrobial enhancer that reduced the CIP dosage by 47% while achieving a better efficacy. The results here again confirmed that an antimicrobial stress is needed for D-tyr^29^. The combination of 80 ppm CIP + 5 ppm D-tyr + 1000 ppm D-ala led to more sessile cells compared with the combination of 80 ppm CIP + 5 ppm D-tyr.

### CLSM observations in the PA biofilm removal test

**Figure 7:**
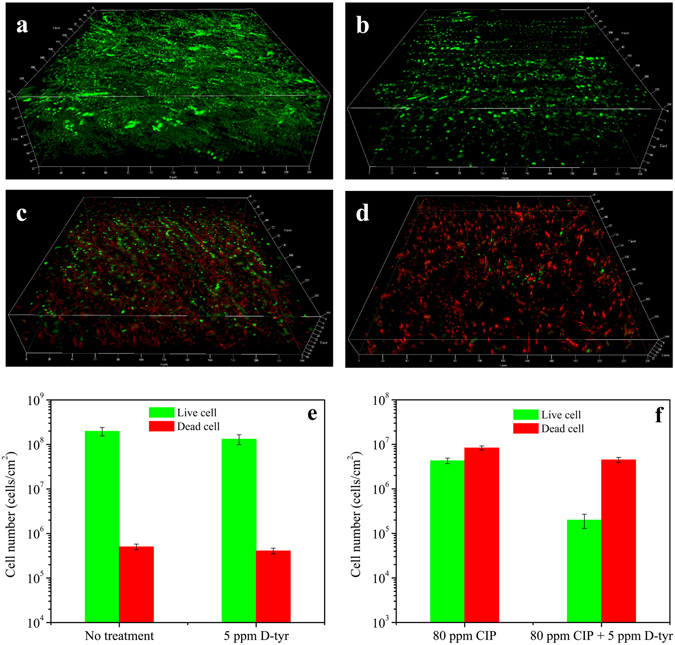
CLSM images of PA biofilms (Dimensions: X = 224 µm, Y = 224 µm) after the 3-hour biofilm removal test in the pH 7.4 PBS buffer containing: (**a**) no treatment (control) (biofilm thickness = 72 µm), (**b**) 5 ppm D-tyr (biofilm thickness = 69 µm), (**c**) 80 ppm CIP (biofilm thickness = 49 µm), and (**d**) 80 ppm CIP + 5 ppm D-tyr (biofilm thickness = 28 µm). The calculated numbers of live/dead cells are shown in (**e**) and (**f**). (Error bars represent standard deviations, statistical reference point n = 6).

The CLSM images of PA biofilms with different treatments are shown in Fig. 7. Live cells were abundant on the no treatment control, and the 5 ppm D-tyr alone treatment coupon surfaces as shown in Fig. 7(a,b). With the 80 ppm CIP alone treatment, there were many dead cells among the live cells (Fig. 7c). With the treatment of 80 ppm CIP + 5 ppm D-tyr (Fig. 7d), fewer live cells were observed than that in the 80 ppm CIP alone treatment. Figure 7e and f show the numbers of live and dead sessile cells for the control, 5 ppm D-tyr, 80 ppm CIP, and 80 ppm CIP + 5 ppm D-tyr treatments after the 3-hour biofilm removal test. They indicate that D-tyr alone had a limited effect to disperse the PA biofilm. The combination of 80 ppm CIP + 5 ppm D-tyr showed better efficacy than the 80 ppm CIP alone treatment. The combination yielded a significant decrease in the sessile cell amount compared with the 80 ppm CIP alone treatment (p = 0.002). The CLSM images corroborated the sessile cell count data well in Fig. 6. The CLSM images show that 5 ppm D-tyr was able to reduce the CIP dosage.

## Discussion

**Figure 8:**
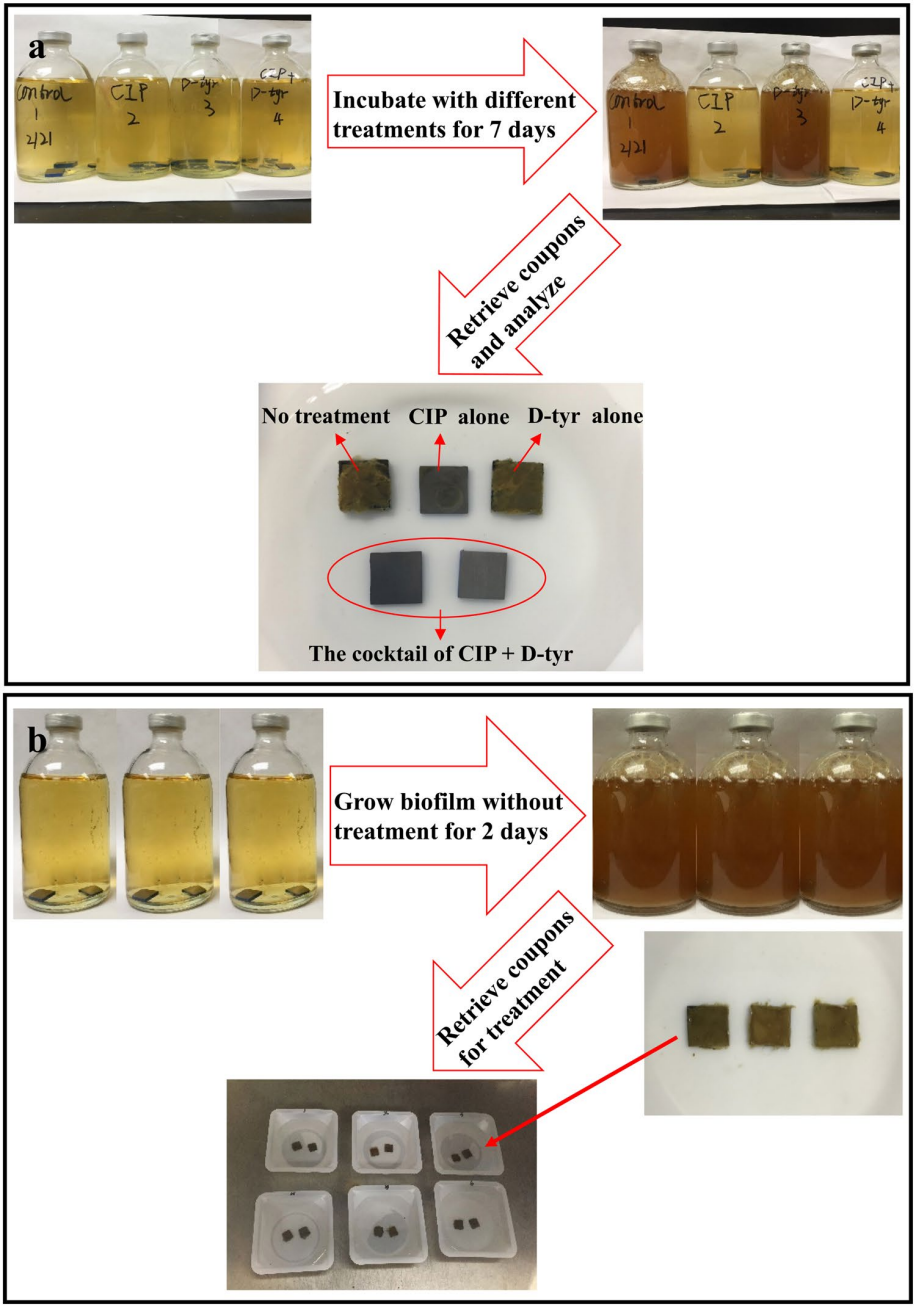
Procedures of the PA biofilm mitigation tests: (**a**) the 7-day biofilm prevention test in anaerobic vials, and (**b**) the 3-hour biofilm removal test in the anaerobic chamber.

Both SEM and CLSM are essential tools to study biofilms. SEM shows the biofilm morphology and corrosion products. CLSM detects the live and dead sessile cells in biofilms without an ability of revealing cell morphology. In the PA biofilm prevention test, SEM was able to show the differences between different treatments. This was because chemicals were added at the beginning with microbes. In Fig. 8a, different coupon surfaces were obtained after 7 days of incubation with different treatments. The no treatment control and the 2 ppm D-tyr alone treatment coupon surfaces were found to be covered by biofilms and corrosion products. While coupons incubated with the cocktail of 30 ppm CIP + 2 ppm D-tyr did not show obvious corrosion products. The sessile cell densities of different treatments on the coupon surfaces were detected by SEM. However, in the biofilm removal test, biofilms were pre-grown to achieve maturity and then treated with different treatments. The killed sessile cells after the short-term (3 hours) biofilm removal test could still remain in the biofilm matrix as shown on the coupon surfaces in Fig. 8b. Under SEM, coupons from different treatments would all show abundant sessile cells because SEM cannot distinguish live cells from the recently killed cells^28^. Thus, SEM would not work well in the biofilm removal test, unless the recently kills cells already detached from the biofilm matrix.

Sarkar & Piresd tested a diverse set of D-amino acids at millimolar concentrations and found they did not inhibit biofilm formation of *B. subtilis*, *S. aureus*, and *Staphylococcus epidermis* ^34^. Recently, Kao *et al*. evaluated the effect of D-alanine, D-leucine, D-methionine, D-tryptophan, and D-tyr at millimolar concentrations on biofilm formation of *P. aeruginosa* (PAO1 and PA14). They found D-amino acids only slow down the biofilm formation rather than completely prevent the biofilm formation^27^. They also concluded that D-amino acid is not an option for potential clinically therapeutic interventions^27^. In this work, D-tyr alone at 2–5 ppm also showed only a limited effect to the PA biofilm prevention (Fig. 1a) and removal (Fig. 6). In the 2 ppm D-tyr alone treatment, there was no statistically significant (p = 0.115) decrease in sessile cell amount compared with the control in the biofilm prevention. Five ppm D-tyr alone slightly reduced the sessile cell amount compared with the control, although the reduced amount was within 1 log. Thus an antimicrobial stress is necessary to improve biofilm dispersal efficacy using D-amino acids^10^ when treating recalcitrant biofilms. This may clarify the confusion in the literature that sometimes a D-amino acid was found effective and sometimes not.

Zilm *et al*. reported that D-amino acids were effective in disrupting *Enterococcus faecalis* biofilms in the presence of sub minimum inhibitory concentrations of antimicrobial^35^. They found that antimicrobials alone did not significantly reduce the optical density of the biofilm samples, while D-amino acids were able to reduce *E. faecalis* biofilms significantly when added together with antimicrobials^35^. Several antibiotics were found by another research group to be enhanced by D-amino acids against an aerobic PA biofilm in polystyrene 96-well plates^11^. The results in this work also demonstrated that increased D-tyr concentration in the combination showed better efficacy. In the PA biofilm prevention test, 1 ppm D-tyr was not sufficient to enhance 30 ppm CIP but 2 ppm D-tyr was found sufficient. D-tyr at 2 ppm enhanced 30 ppm CIP by achieving a similar efficacy compared with the 80 ppm CIP alone treatment in the 7-day biofilm prevention test. In the PA biofilm removal test, the cocktail of 80 ppm CIP + 5 ppm D-tyr achieved extra 1.5-log reduction of sessile cell count compared with the cocktail of 80 ppm CIP + 2 ppm. The cocktail of 80 ppm CIP + 5 ppm D-tyr achieved better efficacy than the 150 ppm CIP alone treatment in the 3-hour biofilm removal test.

The biofilm dispersal mechanisms used by D-amino acids have not been fully ellucidated^36^. In this work, different combinations of treatment chemicals including CIP, D-tyr, and D-ala were performed in the anaerobic PA biofilm prevention and removal tests to corroborate a hypothesis in the literature^25^ that substitution of the D-ala terminus in the bacterial cell walls’ peptidoglycan molecules lead to biofilm dispersal. Such a substitution is said to influence the remodeling of bacteria cell walls^37, 38^. Experimental results in this work show that the addition of 1,000 ppm D-ala reduced the anti-biofilm efficacies of the combinations of 30 ppm CIP + 2 ppm D-tyr in the biofilm prevention test and 80 ppm CIP + 5 ppm D-tyr in the biofilm removal tests. The overwhelmingly high concentration of D-ala in the treatment solutions probably prevented the D-ala terminus from being substituted by D-tyr. This result is consistent with the finding by Xu *et al*. that the addition of a high concentration D-ala to the culture medium counteracted the anti-biofilm effect of the combination of THPS + D-methionine against the *D. vulgaris* biofilm on carbon steel^39^.

In summary, this work found that D-tyr at low concentrations (2–5 ppm) enhanced CIP in both anaerobic PA biofilm prevention and removal tests by achieving similar or better efficacies than higher concentrations of CIP. There was a synergy between D-tyr and CIP, making D-tyr an effective antimicrobial enhancer. Further research is desired to perform efficacy tests in simulated clinical environments.

## Materials and Methods

### Bacterial strain, culture medium, coupons, and chemicals

The wild-type *P. aeruginosa* (PAO1) used in this work was donated by Prof. Daniel J. Hassett of University of Cincinnati College of Medicine. The Luria-Bertani medium with KNO_3_ (LB-NO_3_ medium) was used to grow the bacterium as an NRB. The culture medium pH was adjusted to 7.0 using a sodium hydroxide solution. The culture medium, vials and supplies such as tweezers and pipette tips were autoclaved at 121 °C for 20 min. All liquid solutions were sparged with filter-sterilized nitrogen gas for at least one hour to remove dissolved oxygen. L-cysteine (100 ppm), as an oxygen scavenger, was added to the culture medium to assure the strictly anaerobic condition. An anaerobic chamber filled with N_2_ was used for all anaerobic manipulations. The chamber was disinfected with UV for at least one hour before use. Square-shaped C1018 (UNS G10180) carbon steel coupons with 1 cm^2^ top exposed corrosion surface area were used in the vials. The other surfaces were coated with inert Teflon. The composition of carbon steel was (wt%): C 0.14–0.20, Mn 0.60–0.90, P 0.04, S 0.05, Si 0.15–0.30, and balance Fe. The top coupon surface was abraded with 180, 400, and 600 grit sandpapers sequentially. After that, they were rinsed with 100% isopropanol and air dried under the UV light. CIP and D-tyr were purchased from Sigma-Aldrich (St. Louis, MO, USA). All the other chemicals used in this work were from Fisher Scientific (Pittsburgh, PA, USA) or Sigma-Aldrich.

### Prevention of PA biofilm establishment

For the biofilm prevention test, coupons, PA, the culture medium, treatment chemicals were added together at the beginning upon inoculation. In the anaerobic chamber, 100 ml LB-NO_3_ medium with and without treatment chemicals, 2 ml anaerobic PA seed culture, and 5 replicate coupons were added into 125 ml anaerobic vial. The initial planktonic cell count after inoculation was between 10^5^–10^6^ cells/ml. The anaerobic vials were incubated in a 37 °C incubator (Model 3956, Forma Scientific, OH, USA) without shaking for 7 days. Afterwards, coupons were taken out for sessile cell counting, biofilm observation, and corrosion analysis. This biofilm prevention test was repeated at least twice for accuracy. The procedure is illustrated in Fig. 8a.

### Removal of established PA biofilm

For the biofilm removal test, the LB-NO_3_ medium was used to grow PA biofilms on coupons at 37 °C for 2 days to achieve mature biofilms. Afterwards, the coupons were retrieved and rinsed with a pH 7.4 phosphate buffered saline (PBS) solution to remove the culture medium and planktonic cells in the anaerobic chamber. Then, coupons with biofilms were placed into weighing boats filled with different treatment chemicals for 3 hours in the anaerobic chamber. Each weighing boat with 50 ml pH 7.4 PBS solution contained two coupons. After the 3-hour treatment, coupons were retrieved for sessile cell counting and biofilm observation. This biofilm removal test was repeated at least twice for accuracy. The procedure is illustrated in Fig. 8b.

### Sessile cell counting

The most probable number (MPN) method was adopted to enumerate sessile cells. The MPN liquid culture medium for NRB was purchased from Biotechnology Solutions (Houston, TX, USA). After the biofilm prevention and the biofilm removal tests, the retrieved coupons were first rinsed in the pH 7.4 PBS solution to remove the residual culture medium, planktonic cells, and treatment chemicals. After that, coupons were placed in a 10 ml pH 7.4 PBS solution in a Petri dish. The biofilm on each coupon surface was removed with a small brush. Then, the brush, the coupon, and the 10 ml PBS solution were put in a 50 ml test tube. The test tube was vortexed for 30 s to distribute sessile cells evenly in the PBS solution. Finally, the solution was serially diluted in the 10 ml anaerobic vials and incubated at 37 °C. This cell counting process was repeated twice for accuracy in each test. The t-test statistical method was used to analyze sessile cell counts to provide the p-values for statistical significance.

### Biofilm observations

The biofilm morphology on a coupon surface was observed using a Model JSM-6390 SEM machine (JEOL, Tokyo, Japan). Before the biofilm observation, the biofilm on a coupon was treated with a 4% (w/w) glutaraldehyde solution for 2 hours to fix the biofilm. After that, the biofilm was sequentially dehydrated using different concentrations of isopropanol and then dried using supercritical CO_2_. Finally, the biofilm on the coupon was coated with a conductive palladium film for SEM observation. The detailed biofilm preparation procedure was described previously^28^.

Live and dead cells on a coupon surface were analyzed using a CLSM machine (Model LSM 510, Carl Zeiss, Jena, Germany) based on the procedure described before^28^. Before observation, biofilms were first rinsed with the pH 7.4 PBS solution and then stained with SYTO 9 and propidium iodide (Life Technologies, Grand Island, NY, USA). The numbers of live and dead sessile cells were quantified using the ImageJ software (National Institutes of Health, Bethesda, MD, USA). For both SEM and CLSM observations, the entire coupon surface was checked and a representative image was chosen.

### Corrosion analyses

Corrosion analyses were carried out to evaluate the outcome of PA biofilm treatment. The corrosion test was skipped in the biofilm removal test. This was because the biofilm treatment lasted only 3 hours. The difference of corrosion before and after this short treatment time was negligible. Each weight loss data point was the average of a minimum of 4 coupons after the 7-day biofilm prevention test. The 7-day biofilm prevention test was repeated at least twice. Coupons were weighed before and after the biofilm prevention test. After the 7-day biofilm prevention test, the Clark’s solution was used to remove biofilms and corrosion products from coupon surfaces according to ASTM G1–03^40^. The impact of the acidic Clark’s solution on weight loss was found negligible because the short exposure time of 30 s was short^39^. After biofilms and corrosion products were removed, the pit morphology on a coupon was observed under SEM. The maximum pit depth for each coupon after the 7-day incubation was observed using an infinite focus microscope (IFM) (Model ALC13, Alicona Imaging GmbH, Graz, Austria).

## References

[1] Fonder MA (2008). Treating the chronic wound: A practical approach to the care of nonhealing wounds and wound care dressings. J. Am. Acad. Dermatol..

[2] Dowd SE (2008). Survey of bacterial diversity in chronic wounds using Pyrosequencing, DGGE, and full ribosome shotgun sequencing. BMC Microbiol..

[3] Zhao G (2010). Delayed wound healing in diabetic (db/db) mice with *Pseudomonas aeruginosa* biofilm challenge: a model for the study of chronic wounds: A biofilm-challenged chronic wound model. Wound Repair Regen..

[4] Kirchhoff L (2017). Biofilm formation of the black yeast-like fungus *Exophiala dermatitidis* and its susceptibility to antiinfective agents. Sci. Rep..

[5] Ghanbari A (2016). Inoculation density and nutrient level determine the formation of mushroom-shaped structures in *Pseudomonas aeruginosa* biofilms. Sci. Rep..

[6] Parsek MR, Singh PK (2003). Bacterial biofilms: an emerging link to disease pathogenesis. Annu. Rev. Microbiol..

[7] Johnson RJ, Jurawan I, Frenzel M, Price AC (2016). The identification and mechanism of a *Scenedesmus* spp. causing bio-fouling of an oil field produced water treatment plant. Int. Biodeterior. Biodegrad..

[8] Krishnamurthy A (2015). Superiority of graphene over polymer coatings for prevention of microbially induced corrosion. Sci. Rep..

[9] Li H (2016). Microbiologically influenced corrosion of 2707 hyper-duplex stainless steel by marine *Pseudomonas aeruginosa* biofilm. Sci. Rep..

[10] Li Y, Jia R, Al-Mahamedh HH, Xu D, Gu T (2016). Enhanced biocide mitigation of field biofilm consortia by a mixture of D-amino acids. Front. Microbiol..

[11] Sanchez CJ (2014). D-amino acids enhance the activity of antimicrobials against biofilms of clinical wound isolates of *Staphylococcus aureus* and *Pseudomonas aeruginosa*. Antimicrob. Agents Chemother..

[12] San NO, Nazır H, Dönmez G (2014). Microbially influenced corrosion and inhibition of nickel–zinc and nickel–copper coatings by *Pseudomonas aeruginosa*. Corros. Sci..

[13] Pusic P (2016). Cross-regulation by CrcZ RNA controls anoxic biofilm formation in *Pseudomonas aeruginosa*. Sci. Rep..

[14] Lyczak JB, Cannon CL, Pier GB (2000). Establishment of *Pseudomonas aeruginosa* infection: lessons from a versatile opportunist. Microbes Infect..

[15] Yoon SS (2002). Pseudomonas aeruginosa anaerobic respiration in biofilms: relationships to cystic fibrosis pathogenesis. Dev. Cell.

[16] Widmer AF (2001). New developments in diagnosis and treatment of infection in orthopedic implants. Clin. Infect. Dis..

[17] Lou Y (2016). Antibacterial ability of a novel Cu-bearing 2205 duplex stainless steel against *Pseudomonas aeruginosa* biofilm in artificial seawater. Int. Biodeterior. Biodegrad..

[18] Mansouri H, Alavi SA, Fotovat M (2015). Microbial-influenced corrosion of corten steel compared with carbon steel and stainless steel in oily wastewater by *Pseudomonas aeruginosa*. JOM.

[19] Abdolahi A, Hamzah E, Ibrahim Z, Hashim S (2014). Microbially influenced corrosion of steels by Pseudomonas aeruginosa. Corros. Rev..

[20] Manam NS (2017). Study of corrosion in biocompatible metals for implants: A review. J. Alloys Compd..

[21] Li LM, Wong T, Rose E, Wickham G, Bryce E (2016). Evaluation of an ultraviolet C light–emitting device for disinfection of electronic devices. Am. J. Infect. Control.

[22] Mah T-FC, O’Toole GA (2001). Mechanisms of biofilm resistance to antimicrobial agents. Trends Microbiol..

[23] Chalhoub H (2017). Mechanisms of intrinsic resistance and acquired susceptibility of *Pseudomonas aeruginosa* isolated from cystic fibrosis patients to temocillin, a revived antibiotic. Sci. Rep..

[24] Xu D, Jia R, Li Y, Gu T (2017). Advances in the treatment of problematic industrial biofilms. World J. Microbiol. Biotechnol..

[25] Kolodkin-Gal I (2010). D-amino acids trigger biofilm disassembly. Science.

[26] Friedman M (1999). Chemistry, Nutrition, and Microbiology of D-Amino Acids. J. Agric. Food Chem..

[27] Kao WTK, Frye M, Gagnon P, Vogel JP, Chole R (2017). D-amino acids do not inhibit *Pseudomonas aeruginosa* biofilm formation. Laryngoscope Investig. Otolaryngol..

[28] Jia R, Yang D, Li Y, Xu D, Gu T (2017). Mitigation of the *Desulfovibrio vulgaris* biofilm using alkyldimethylbenzylammonium chloride enhanced by D-amino acids. Int. Biodeterior. Biodegrad..

[29] Xu D, Li Y, Gu T (2012). A synergistic D-tyrosine and tetrakis hydroxymethyl phosphonium sulfate biocide combination for the mitigation of an SRB biofilm. World J. Microbiol. Biotechnol..

[30] Hamzah E, Hussain MZ, Ibrahim Z, Abdolahi A (2013). Influence of *Pseudomonas aeruginosa* bacteria on corrosion resistance of 304 stainless steel. Corros. Eng. Sci. Technol..

[31] Lan G (2017). Corrosion of carbon steel induced by a microbial-enhanced oil recovery bacterium *Pseudomonas* sp. SWP-4. RSC Adv.

[32] Jia R, Yang D, Xu D, Gu T (2017). Electron transfer mediators accelerated the microbiologically influence corrosion against carbon steel by nitrate reducing *Pseudomonas aeruginosa* biofilm. Bioelectrochemistry.

[33] Xu D, Li Y, Song F, Gu T (2013). Laboratory investigation of microbiologically influenced corrosion of C1018 carbon steel by nitrate reducing bacterium *Bacillus licheniformis*. Corros. Sci..

[34] Sarkar S, Pires MM (2015). D-amino acids do not inhibit biofilm formation in *Staphylococcus aureus*. PloS One.

[35] Zilm PS (2017). D-amino acids reduce *Enterococcus faecalis* biofilms *in vitro* and in the presence of antimicrobials used for root canal treatment. PloS One.

[36] Jia R, Yang D, Al-Mahamedh HH, Gu T (2017). Electrochemical testing of biocide enhancement by a mixture of D-amino acids for the prevention of a corrosive biofilm consortium on carbon steel. Ind. Eng. Chem. Res.

[37] Cava F, Lam H, de Pedro MA, Waldor MK (2011). Emerging knowledge of regulatory roles of D-amino acids in bacteria. Cell. Mol. Life Sci..

[38] Lam H (2009). D-Amino acids govern stationary phase cell wall remodeling in bacteria. Science.

[39] Xu D, Li Y, Gu T (2014). D-methionine as a biofilm dispersal signaling molecule enhanced tetrakis hydroxymethyl phosphonium sulfate mitigation of *Desulfovibrio vulgaris* biofilm and biocorrosion pitting. Mater. Corros..

[40] Zhang P, Xu D, Li Y, Yang K, Gu T (2015). Electron mediators accelerate the microbiologically influenced corrosion of 304 stainless steel by the *Desulfovibrio vulgaris* biofilm. Bioelectrochemistry.

